# Pain severity trajectory and influencing factors in children with recurrent aphthous stomatitis: a latent variable growth mixture model

**DOI:** 10.3389/fpain.2026.1869523

**Published:** 2026-07-07

**Authors:** Hu Ruping, Song Hongjie

**Affiliations:** Department of Stomatology, Chengdu Second People’s Hospital, Chengdu, Sichuan Province, China

**Keywords:** children, influencing factors, pain intensity, progression, recurrent aphthous stomatitis

## Abstract

**Objective:**

To explore the potential categories of pain intensity development trajectory in children with recurrent aphthous stomatitis and analyze the influencing factors of category differences.

**Methods:**

Conveniently select 200 children with recurrent aphthous stomatitis treated in our hospital from September 2023 to June 2025 as the research subjects, and retrospectively collect the pain dimension scores of the local body sign scores of oral ulcers at the time of visit (T0), as well as on the third day (T1), seventh day (T2), and fourteenth day (T3) after visit. The optimal number of categories was analyzed and named using a latent variable growth mixture model. Clinical data of the children were collected using a self-designed clinical data questionnaire. Independent influencing factors of potential category differences were analyzed by univariate analysis and multivariate logistic regression.

**Results:**

The pain intensity development trajectory of the 200 children with recurrent aphthous stomatitis could be divided into three potential categories: high pain level relief type (68 cases, 34.00%), moderate pain level improvement type (82 cases, 41.00%), and low pain level stable type (50 cases, 25.00%). Univariate and multivariate logistic regression analyses showed that for the high pain relief type, ulcer size, number of ulcers, ulcer type, frequency of attacks, and picky eating are independent influencing factors (*P* < 0.05); For the moderate pain level improvement type, ulcer classification, frequency of attacks, and picky eating are independent influencing factors (*P* < 0.05).

**Conclusion:**

The development trajectory of pain severity in children with recurrent aphthous stomatitis exhibits certain heterogeneity and is influenced by factors such as ulcer size, number of ulcers, ulcer type, frequency of attacks, and picky eating habits. However, there are differences in the influencing factors of different trajectory categories. The above findings can provide reference for identifying high-risk pain trajectory children.

## 1 Introduction

Recurrent aphthous stomatitis (RAS) are one of the most common oral mucosal diseases in children. Their formation is closely related to a variety of factors such as abnormal immune regulation, genetic susceptibility and nutritional status. These factors may lead to local mucosal inflammation and epithelial barrier damage (1). Clinically, it manifests as single or multiple painful ulcers, accompanied by a surrounding erythematous halo and pseudomembrane coverage, which seriously affects the child's eating, swallowing and speech functions (2, 3). With the advancement of pediatric diagnosis and treatment technology, symptomatic treatments such as antibacterial/anti-inflammatory mouthwash, anti-inflammatory drugs and immunomodulators such as corticosteroids have been widely used in clinical practice. Due to their advantages of effectively relieving pain and promoting ulcer healing, they have become a commonly used treatment method in clinical practice (4). However, RAS is characterized by self-limitation but recurrent attacks. Some patients experience severe pain and slow relief, which has a significant negative impact on their physical and mental health and quality of life (5). Although domestic research has begun to focus on the pain problem of patients with RAS, most studies only focus on the pain score of a single attack or cross-sectional analysis, and the research subjects are mostly adult patients. There is insufficient exploration of the pain characteristics and tolerance differences of children as a special group (6). Furthermore, there is currently a lack of in-depth analysis of the development trajectory of pain intensity in children with RAS based on longitudinal time series data. Therefore, this study collected pain score data at four time points from children with RAS treated at our hospital. A latent variable growth mixture model was used to classify the development trajectory of pain intensity. Univariate analysis and multivariate logistic regression analysis were conducted to analyze the influencing factors of differences in pain trajectory, aiming to provide a theoretical basis for identifying children with high-risk pain trajectories, developing personalized interventions, and improving RAS pain management in children.

## 2 Materials and methods

This study is a retrospective longitudinal study. All data are from the medical records of children with RAS who received treatment at our hospital between September 2023 and June 2025. The researchers retrospectively extracted pain scores at four predetermined time points (admission, day 3, day 7, and day 14) from medical records and retrieved clinical features from the same source.

### 2.1 Calculation of sample size

According to Kendall's principle, the sample size is 10 to 20 times the number of independent variables (7). In this study, there are 12 independent variables. Considering a 10% sample loss rate, the sample size is calculated to be 132 to 264 cases. In addition, using G * Power 3.1 software for *post hoc* efficacy analysis, for multi class logistic regression (with OR = 1.5 and *α* = 0.05), the efficacy provided by the current sample size (*n* = 200) exceeds 0.85. According to the sample size recommendation of the latent variable growth mixed model, at least 10–20 cases are required for each category. In this study, there were 68 cases, 82 cases, and 50 cases for the three categories, which meet the requirements. Based on the actual number of patients in our hospital, this study finally selected 200 children with RAS treated in our hospital from September 2023 to June 2025 as the research subjects using convenience sampling.

### 2.2 Inclusion and exclusion criteria

Inclusion criteria: (1) meeting the diagnostic criteria for RAS (8); (2) age ≤ 18 years; (3) complete clinical data.

Exclusion criteria: (1) Having two or more oral mucosal diseases at the same time; (2) Insufficient function of vital organs such as heart, liver, and kidney; (3) History of acute infection and recent surgery; (4) Having taken systemic drugs, vitamin or mineral supplements or immunosuppressive drugs in the past 6 months; (5) Having other chronic pain diseases; (6) Having mental disorders or impaired consciousness.

### 2.3 Data collection

#### 2.3.1 Pain intensity analysis

Based on routine clinical follow-up nodes and the natural course of oral ulcers, pain levels were evaluated at the time of patient visit (T0), acute inflammatory phase on the third day after visit (T1), early healing phase on the seventh day (T2), and complete healing or recovery phase on the fourteenth day (T3). The pain level of the patient was quantitatively assessed using the pain dimension of the local signs score for oral ulcers (9). It is a specialized assessment tool in the field of oral mucosal diseases, and the pain dimension and local signs together constitute a complete disease assessment system with good content validity. This rating is based on the functional evaluation criteria of influencing eating, which facilitates the response and clinical recording of young children. And based on retrospective design, this score is a routine recording item in our hospital's medical records, which can ensure the consistency of data extraction. The score divides the pain level into 4 levels: 0 points = no pain, 1 point = mild pain, 2 points = significant pain but not affecting eating, and 3 points = significant pain and affecting eating. The higher the score, the more severe the pain level of the patient. Pain scores at each time point were extracted based on the patient's chief complaint and physical examination record in the medical record. If there were multiple records at the same time point, the pain score officially recorded by the doctor at the time of the visit was used. As a retrospective study, not all patients had medical records on the precise 3rd, 7th, and 14th day. For cases where there are fluctuations in the consultation time, they will be uniformly classified into the nearest standard time point for analysis.

#### 2.3.2 Clinical data collection

By reviewing the literature, the research team designed their own clinical data questionnaire to collect information including age, gender, ulcer size, number of ulcers, ulcer type, ulcer location, frequency of attacks, white blood cell count, family history, allergy history, whether the patient is a picky eater, and whether they use hormones/antibiotics (see Supplementary File). To ensure the relevance and comprehensiveness of the items, an expert group consisting of three associate chief dentists and two senior pediatric nursing experts was invited to evaluate the content validity of the questionnaire. The expert evaluation results show that the item level content validity index (I-CVI) is 0.83–1.00, and the average content validity index (S-CVI/Ave) of the questionnaire is 0.94, indicating that the questionnaire content has good representativeness. Before the formal investigation, two trained researchers independently pre extracted 20 pre selected medical records. The consistency of the two researchers’ judgments on subjective variables such as picky eating has a Kappa value of 0.85 (95% CI: 0.72∼0.94), and the consistency of extracting objective clinical indicators is 100%, indicating good inter rater reliability. Picky eating is defined as a pattern of limited dietary diversity, persistent avoidance of new foods (food neophobia), and refusal to eat conventional foods in typical developing children. Whether children are picky eaters is based on parental reports during the medical history collection period, and this variable is evaluated as a binary indicator (yes/no). If parents acknowledge that their child continues to exhibit these behaviors (for more than 3 months) to the extent that they affect their parents or child's dietary intake, the child is classified as a picky eater.

### 2.4 Statistical methods

Mplus 8.3 software was used to construct a latent variable growth mixture model. This study adopts a linear latent variable growth mixed model. The slope factor load is encoded as 0, 3, 7, and 14 based on the actual number of days. The model equation is: Pain_it = *η*_0i + *η*_1i × Time_t + *ε* it. Among them, *η* 0i (random intercept) represents the initial pain level of the child at T0, *η* 1i (random slope) represents the linear rate of change of pain over time (unit: points/day), and *ε* it is the residual. The intra class variance is set as homogeneous, meaning that the residual variances within each potential category are assumed to be equal. The intercept and slope allow for covariance. The model estimation adopts the robust maximum likelihood estimation method (MLR). To avoid getting stuck in local optima, use 500 random starts and 100 final optimizations. The missing data processing adopts the full information maximum likelihood method (FIML), assuming that the data is randomly missing. Smaller Akaike Information Criterion (AIC), Bayesian Information Criterion (BIC), and sample-corrected Bayesian Information Criterion (aBIC) values indicate better model fit. Entropy values closer to 1 indicate more accurate model classification. A Bootstrap-based likelihood ratio (BLRT) test result <0.05 indicates that the model with this number of trajectory categories performs better than the model with the number of trajectory categories minus 1. Based on model fit indices and clinical interpretability, the optimal number of trajectory categories was determined.

SPSS 21.0 software was used for statistical analysis. Qualitative data are expressed as the number of cases and percentages (%), and inter-group comparisons were performed using the *χ^2^* test. For variables with *P* < 0.05 in univariate analysis, further pairwise comparisons between the three groups were conducted using the Bonferroni method. Quantitative data conforming to a normal distribution were expressed as mean ± standard deviation ($\bar{x}$ ± *s)*, and one-way ANOVA was used for comparisons among multiple groups. Quantitative data not conforming to a normal distribution were expressed as median (interquartile range) [*M* (*P* 25, *P* 75)], and Kruskal–Wallis H test was used for comparisons among multiple groups. This study used pain score (0–3 points) as a continuous variable for main analysis, for the following reasons: (1) This scale is often treated as a continuous variable in previous studies on oral mucosal diseases; (2) Each level is distinguished by the criterion of “whether it affects eating”, and clinical differences have practical significance, approximately satisfying the equidistant hypothesis; (3) Treating ordered variables as continuous variables can improve statistical efficiency and facilitate the convergence of LGMM models. To test the robustness of the results, we also conducted sensitivity analysis, using pain score as an ordered categorical variable and reanalyzing it using WLSMV estimation method.

Using the pain intensity trajectory categories identified by the latent variable growth mixed model as the dependent variable, multivariate multivariate logistic regression analysis was used to analyze the influencing factors of the pain intensity trajectory in children with RAS. Before conducting regression analysis, multicollinearity diagnosis was performed, and the variance inflation factors (VIFs) were all less than 5, indicating the absence of significant collinearity. A *p-*value < 0.05 was considered statistically significant.

## 3 Results

### 3.1 Analysis of the current status of pain intensity in children with recurrent aphthous stomatitis

Analysis of variance showed that the pain scores of children in T0 to T3 were (1.67 ± 1.07), (1.37 ± 0.87), (0.92 ± 0.79), and (0.61 ± 0.62), respectively, *F* = 192.208, *P* < 0.001.

### 3.2 Potential categories of pain intensity development trajectory in children with recurrent aphthous stomatitis

Before determining the number of potential categories, a comparison was made between linear and quadratic growth functions. Considering that the four time points are designed unevenly (0, 3, 7, 14 days), and the linear model is more concise and clinically interpretable, a linear growth function was ultimately chosen as the basis for the latent variable growth mixed model.

Using pain scores at four time points (T0-T3) in children with RAS as the explicit indicator, 1–5 latent class models were established. The optimal latent class model was evaluated based on AIC, BIC, aBIC, and BLRT tests, and the results are shown in Table 1. As the number of classes increased, AIC, BIC, and aBIC gradually decreased. When there were 3 classes, Entropy = 0.846 > 0.800, LRT = 0.022 < 0.05, and BLRT = 0.018 < 0.05, with each class accounting for >10%. When there were 4 classes, AIC, BIC, and aBIC further decreased, but LRT = 0.124 > 0.05 and BLRT = 0.118 > 0.05, indicating that the 4-class model was not significantly better than the 3-class model. Considering both model fit indices and clinical interpretability, the 3-class model was ultimately selected as the optimal model. The average posterior probabilities for each category are 0.92 (category 1), 0.89 (category 2), and 0.94 (category 3), indicating high classification accuracy.

**Table 1:** Potential categories of pain intensity development trajectory in children with recurrent aphthous stomatitis.

Number of categories	AIC	BIC	aBIC	Entropy	LRT	BLRT	Category percentage
1	925.772	949.219	920.795				
2	887.380	918.642	880.743	0.857	0.038	0.041	0.42/0.58
3	809.456	848.534	833.251	0.846	0.022	0.018	0.41/0.34/0.25
4	786.615	833.508	776.659	0.812	0.124	0.118	0.35/0.28/0.22/0.15
5	766.988	821.696	755.373	0.791	0.356	0.342	0.31/0.26/0.20/0.14/0.09

### 3.3 Pain intensity classification and naming

**Figure 1:**
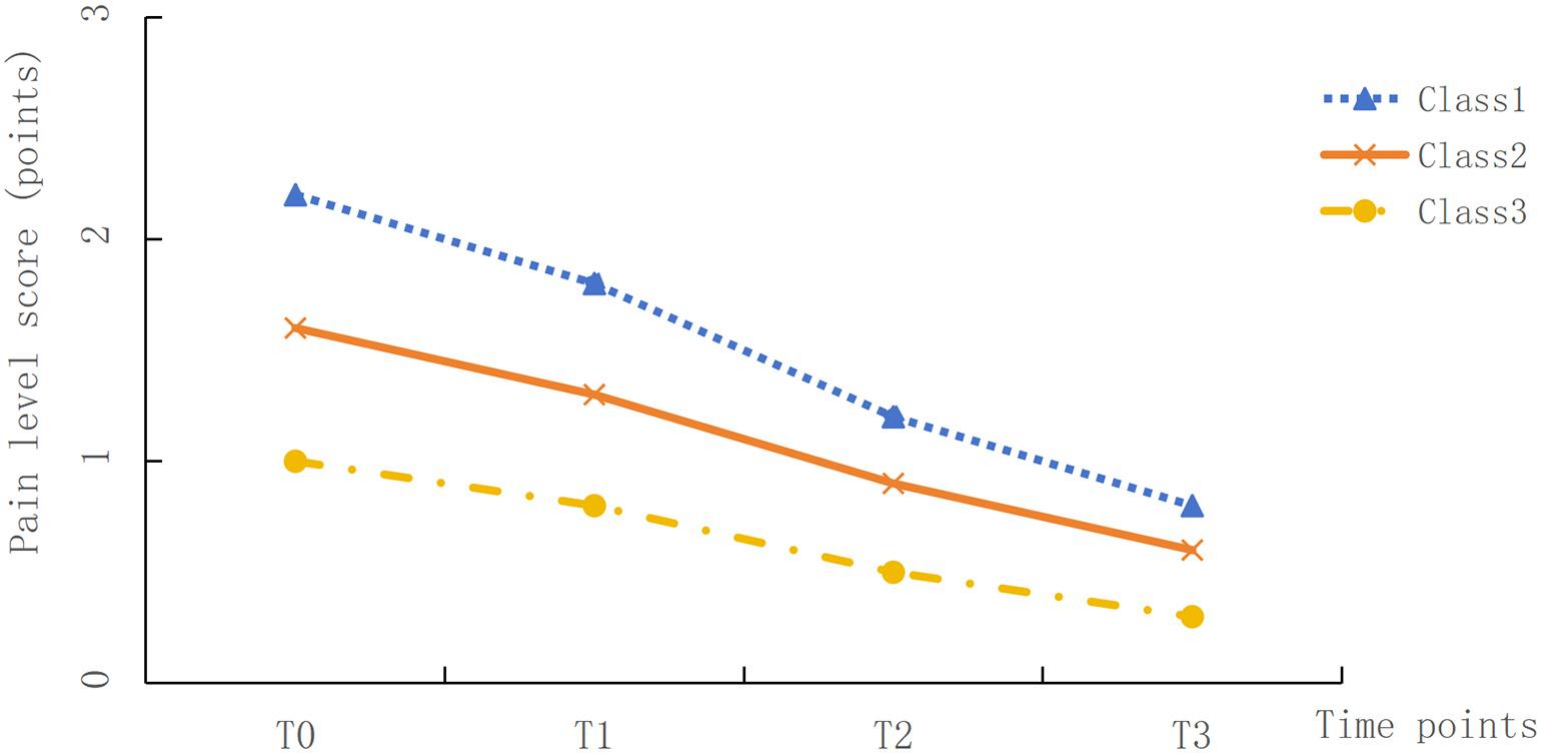
Potential category map of the development trajectory of pain intensity in children with recurrent aphthous stomatitis.

Based on a three-category model, a trajectory diagram of potential categories was plotted with time points as the *x*-axis and the pain intensity score of RAS in children as the *y*-axis, as shown in Figure 1. Category 1, comprising 68 cases (34%), showed high pain intensity at T0, which gradually decreased over time, and can be considered as high-pain-level relief type; Category 2, comprising 82 cases (41%), showed moderate pain intensity at T0, which showed a slow decreasing trend over time, and can be considered as moderate-pain-level improvement type; Category 3, comprising 50 cases (25%), showed low pain intensity at T0, which did not change much or fluctuated slightly over time, and can be considered as low-pain-level stable type. See Table 2.

**Table 2:** Estimated growth parameters for each potential category.

Potential categories	Mean of intercept (initial pain, score)	Variance of intercept	Mean of slope (rate of change, points/day)	Variance of slope	Average posterior probability	Class proportion (%)
High-pain-level relief type	2.20 (0.12)	0.45 (0.08)	−0.105 (0.015)	0.03 (0.01)	0.92	34.00
Moderate-pain-level improvement type	1.60 (0.10)	0.32 (0.06)	−0.075 (0.012)	0.02 (0.01)	0.89	41.00
Low-pain-level stable type	1.00 (0.08)	0.21 (0.05)	−0.048 (0.010)	0.01 (0.00)	0.94	25.00

### 3.4 Univariate analysis of potential categories of pain severity development trajectory in children with recurrent aphthous stomatitis

Univariate analysis showed that there were statistically significant differences among the three groups in ulcer size, number of ulcers, ulcer type, frequency of attacks, and whether the patient was a picky eater (*P* < 0.05), as shown in Table 3.

**Table 3:** Univariate analysis of potential categories of pain severity development trajectory in children with recurrent aphthous stomatitis.

Influencing factors	High-pain-level relief type (*n* = 68)	Moderate-pain-level improvement type (*n* = 82)	Low-pain-level stable type (*n* = 50)	*χ* ^2^	*P*
age				0.392	0.822
0–12 years old	45 (66.18)	58 (70.73)	35 (70.00)		
13–18 years old	23 (33.82)	24 (29.27)	15 (30.00)		
gender				0.044	0.978
male	36 (52.94)	42 (51.22)	26 (52.00)		
female	32 (47.06)	40 (48.78)	24 (48.00)		
Ulcer size				20.588	<0.001
<5 mm	28 (41.18)^a^	52 (63.41)^b^	41 (82.00)^b^		
≥5 mm	40 (58.82)^a^	30 (36.59)^b^	9 (18.00)^b^		
Number of ulcers				12.293	0.002
1∼2	35 (51.47)^a^	56 (68.29)^a,b^	41 (82.00)^b^		
≥3	33 (48.53)^a^	26 (31.71)^a,b^	9 (18.00)^b^		
Ulcer Classification					
Lightweight	38 (55.88)^a^	50 (60.98)^b^	41 (82.00)^c^	9.338	0.009
Severe/Herpesoid	30 (44.12)^a^	32 (39.02)^b^	9 (18.00)^c^		
Ulcer site				3.221	0.200
Front (lips, cheeks)	38 (55.88)	52 (63.41)	36 (72.00)		
Posterior part (tongue/palatine/pharynx)	30 (44.12)	30 (36.59)	14 (28.00)		
attack frequency				8.536	0.014
≤6 times/year	34 (50.00)^a^	46 (56.10)^b^	38 (76.00)^c^		
>6 times/year	34 (50.00)^a^	36 (43.90)^b^	12 (24.00)^c^		
White blood cell count				0.430	0.806
normal	48 (70.59)	60 (73.17)	38 (76.00)		
rise	20 (29.41)	22 (26.83)	12 (24.00)		
Family history				0.218	0.897
none	41 (60.29)	52 (63.41)	32 (64.00)		
have	27 (39.71)	30 (36.59)	18 (36.00)		
Allergy history				0.875	0.646
none	45 (66.18)	58 (70.73)	37 (74.00)		
have	23 (33.82)	24 (29.27)	13 (26.00)		
Are you a picky eater?				11.128	0.004
no	28 (41.18)^a^	46 (56.10)^a,b^	36 (72.00)^b^		
yes	40 (58.82)^a^	36 (43.90)^a,b^	14 (28.00)^b^		
Whether hormones/antibiotics are used				1.836	0.399
no	30 (44.12)	43 (52.44)	28 (56.00)		
yes	38 (55.88)	39 (47.56)	22 (44.00)		

Univariate analysis is an exploratory screening method, with *P* < 0.05 as the criterion for inclusion in multivariate analysis. For variables with *P* < 0.05 in univariate analysis, further pairwise comparisons between the three groups were conducted using the Bonferroni method. Different letters (a, b, c) in peers indicate statistically significant differences between groups. (Bonferroni corrected *P* < 0.05).

### 3.5 Multivariate logistic regression analysis of potential categories in the development trajectory of pain intensity in children with recurrent aphthous stomatitis

**Figure 2:**
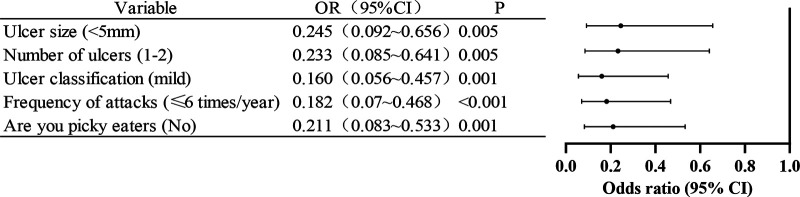
Forest plot of factors related to pain relief at high pain levels.

**Figure 3:**
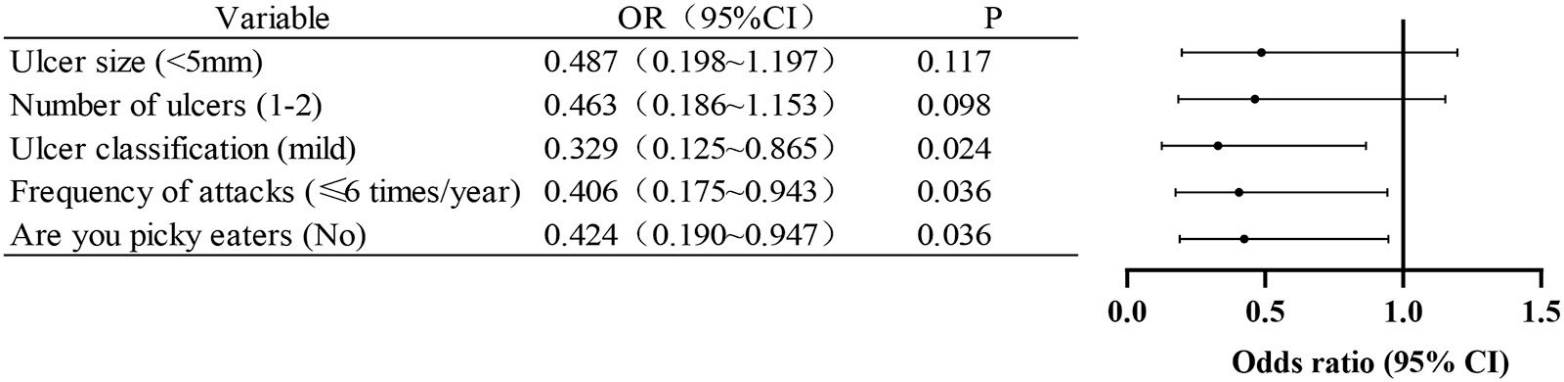
Forest plot of factors related to pain level improvement.

Using the three categories of pain intensity development trajectory as dependent variables, and variables with statistically significant differences in univariate analysis as independent variables, a multivariate logistic regression analysis was conducted with a low-pain-level stable type as a reference. Variable assignments are shown in Table 4. Before conducting regression analysis, the multicollinearity diagnosis showed that the variance inflation factor (VIF) ranged from 1.12 to 1.78, all less than 5, indicating the absence of significant multicollinearity issues. The results showed that for the high pain relief type, ulcer size (<5 mm), number of ulcers (1–2), ulcer type (mild), frequency of attacks (≤6 times/year), and picky eating (no) were all protective factors (OR values < 1, *P* < 0.05z； For the moderate pain level improvement type, ulcer classification (mild), frequency of attacks (≤6 times/year), and picky eating (no) were protective factors (*P* < 0.05), while there was no statistically significant difference in ulcer size and number in this group (*P* = 0.117 and *P* = 0.098). See Table 5. The forest plot visualization of the regression results is shown in Figures 2, 3.

**Table 4:** Variable assignment.

Variable	Assignment
Ulcer size	<5 mm = 0; ≥5 mm =1
Number of ulcers	1∼2 = 0; ≥ 3 = 1
Ulcer Classification	Mild type = 0; Severe/herpetic type = 1
attack frequency	≤6 times/yea*r* = 0; > 6 times/yea*r* = 1
Are you a picky eater?	No = 0; Yes = 1

**Table 5:** Multivariate logistic regression analysis of potential categories in the development trajectory of pain intensity in children with recurrent aphthous stomatitis.

Related factors	B	Standard error	Wald	*P*	OR value	95% CI of OR value	
						lower limit	upper limit
High pain level relief							
Ulcer size (<5 mm)	−1.405	0.502	7.841	0.005	0.245	0.092	0.656
Number of ulcers (1∼2)	−1.456	0.516	7.971	0.005	0.233	0.085	0.641
Ulcer Classification (Mild)	−1.832	0.535	11.705	0.001	0.160	0.056	0.457
Attack frequency (≤ 6 times/year)	−1.706	0.483	12.469	<0.001	0.182	0.070	0.468
Are you a picky eater? (No)	−1.558	0.474	10.797	0.001	0.211	0.083	0.533
Improved pain level							
Ulcer size (<5 mm)	−0.719	0.459	2.457	0.117	0.487	0.198	1.197
Number of ulcers (1∼2)	−0.769	0.465	2.736	0.098	0.463	0.186	1.153
Ulcer Classification (Mild)	−1.111	0.493	5.081	0.024	0.329	0.125	0.865
Attack frequency (≤ 6 times/year)	−0.902	0.430	4.398	0.036	0.406	0.175	0.943
Are you a picky eater? (No)	−0.859	0.410	4.376	0.036	0.424	0.190	0.947

The variables were compared with the control group for “ulcer size (≥ 5 mm), number of ulcers (≥ 3), ulcer type (severe/herpetic), frequency of attacks (> 6 times/year), and picky eating (yes)”. The VIF of multicollinearity diagnosis is all < 5.

### 3.6 Sensitivity analysis

To test the robustness of treating pain score as a continuous variable, we used WLSMV estimation method to re fit LGMM with pain score as an ordered categorical variable (0–3 points). The results showed that the three category model (Entropy = 0.831, BLRT < 0.05) was still supported, and the OR values and significance levels of each influencing factor were basically consistent with the main analysis, indicating that the conclusions of this study have good robustness (see Supplementary Materials for details).

## 4 Discussion

### 4.1 Analysis of the current status of pain intensity in children with recurrent aphthous stomatitis

The results of this study showed that the pain score of 200 children with RAS at the time of consultation (T0) was (1.67 ± 1.07), which was at the level of moderate pain. The score gradually decreased over time, and dropped to (0.61 ± 0.62) at T3. This result suggests that children with RAS suffer from a relatively significant pain burden during the acute phase, but the pain can be effectively relieved after symptomatic treatment or natural disease progression. The reasons for the high initial pain level in children with RAS may be due to the following aspects. First, RAS is essentially an immune inflammatory disease. During an attack, the local mucosa shows a typical erythematous halo and pseudomembrane coverage. Activation of the NLRP3 inflammasome leads to the release of pain-inducing cytokines such as IL−1*β* and TNF-α, which directly stimulate the nerve endings in the submucosa and cause severe pain (10). Secondly, the basal epithelium of children's mucosa is thinner and the cell renewal rate is different from that of adults, making children's oral mucosa more prone to barrier damage when subjected to the same stimulation, and their pain sensitivity during ulcer healing is higher (11). In addition, in this study, children with ulcers ≥5 mm in size, ≥3 ulcers, severe/herpetic type, and an attack frequency >6 times/year were mainly concentrated in the high pain level relief group, suggesting that the clinical severity of ulcers directly determines the intensity of pain in the acute phase. However, although the overall pain level decreased over time, about 34% of the children were still at a high pain level at T0, and their pain reduction rate was relatively slow. It is speculated that these children may also have persistent risk factors such as picky eating habits leading to nutritional deficiencies, resulting in more severe inflammatory response and delayed healing (12, 13). Fang P et al. (14) found that patients with RAS had widespread deficiencies in vitamins B6, B7, C and 25(OH)D3. In this study, picky eaters accounted for 58.82% of the high pain relief group, indirectly proving the important influence of nutritional status on the pain level of RAS. This suggests that healthcare workers need to have a clear understanding of the development trajectory of oral ulcer pain in RAS patients, especially for high pain relief group patients, and need to provide targeted intervention measures to alleviate the pain of patients to the greatest extent.

### 4.2 Factors influencing the increased pain intensity of recurrent aphthous stomatitis in children

The results of this study indicate that ulcer size, number of ulcers, ulcer type, frequency of attacks, and picky eating are all independent influencing factors on the development trajectory of pain intensity in children with RAS (*P* < 0.05). The mechanism may be that ulcer size is one of the core clinical indicators affecting pain intensity. In this study, children with ulcers ≥5 mm accounted for 58.82% of the high pain level relief type, while only 18.00% accounted for the low pain level stable type, and the difference was statistically significant (*P* < 0.05). Larger ulcers are usually accompanied by deeper mucosal defects and denser nerve ending exposure, and the release of more inflammatory mediators will directly activate pain receptors, resulting in a higher initial pain level (15). At the same time, in this study, children with ≥3 ulcers accounted for 48.53% of the high pain level relief type, while 18.00% accounted for the low pain level stable type (*P* < 0.05). Multiple ulcers mean that there are multiple pain sources in the oral cavity, and the spatial superposition effect of pain signals will significantly enhance the overall pain perception (16). In addition, the presence of multiple ulcers may interfere with the child's eating, swallowing and speech functions, thereby affecting the pain relief trajectory. Ulcer subtypes can also predict the pain trajectory of children with RAS. In this study, severe/herpetic ulcers accounted for 44.12% of the high pain level relief type and 18.00% of the low pain level stable type (*P* < 0.05). Severe ulcers have a diameter of more than 1 cm, involve deeper tissue layers, can take up to 30 days to heal and are prone to scarring (17); herpetic ulcers are smaller in diameter but numerous and easy to merge (18). The pathological characteristics of these two subtypes determine that their pain is more severe and lasts longer, so that these children present a trajectory of high pain level and slow relief. Attack frequency is an important indicator of disease activity. In this study, children with an attack frequency of >6 times/year accounted for 50.00% of the high pain level relief type and 24.00% of the low pain level stable type (*P* < 0.05). In children with frequent attacks, the oral mucosa undergoes repeated damage and repair processes, and the local tissues may have a persistent subclinical inflammatory state. The pain receptors are in a state of long-term sensitivity, resulting in more intense pain experience in each attack (19, 20). In addition, the shortened interval between attacks makes it difficult for the pain burden to completely subside. Therefore, children with frequent attacks are more likely to be classified as high pain trajectory. Whether or not one is a picky eater is an important indirect indicator of nutritional status (21). In this study, picky eaters accounted for 58.82% of the high pain level relief type and 28.00% of the low pain level stable type (*P* < 0.05). The study by Nogueira-de-Almeida CA et al. (22) showed that picky eaters may have insufficient intake of B vitamins, vitamin C and vitamin D. Vitamin C is involved in collagen synthesis and the maintenance of basement membrane integrity. Its deficiency can lead to impaired mucosal barrier function and delayed ulcer healing (23); B vitamin deficiency may affect the normal function of the nervous system and the proliferation and differentiation of epithelial cells (24); vitamin D deficiency is closely related to the imbalance of inflammatory regulation (25). These mechanisms work together and may make the pain more severe and the relief slower for picky eaters.

### 4.3 Potential clinical significance of this study

The results of this study indicate that ulcer size, number of ulcers, ulcer type, frequency of attacks, and whether the child is a picky eater are all factors influencing the development trajectory of pain in children with RAS. These results suggest that identifying the above factors may help in early screening of high-risk children belonging to the high pain relief trajectory and provide clues for optimizing pain management strategies. A standardized pain assessment process can be established for this child, and dynamic pain monitoring can be carried out using the local signs score of oral ulcers. Local anesthetics, anti-inflammatory mouthwash, and other symptomatic treatment plans can be selected according to the degree of pain. In addition, nurses can provide targeted disease health education and nutritional guidance to parents and children to help them fully understand the pathogenesis, inducing factors, and daily prevention methods of RAS, as well as how to reduce the frequency of attacks and alleviate the degree of pain by adjusting the diet, such as increasing the intake of foods rich in B vitamins, vitamin C, and vitamin D (26). For children with ulcer size ≥  mm, number of ulcers ≥3, and severe/herpetic type, closer follow-up observation and personalized intervention measures such as photobiomodulation therapy can be considered. Future research can further verify whether shortening the follow-up interval can promote ulcer healing and relieve pain (27). For children with high-frequency attacks with a frequency of more than 6 attacks per year, recording an attack diary to identify potential triggering factors may have certain clinical value, but the impact of this strategy on pain trajectory still needs to be confirmed by prospective studies. For children with picky eating habits, it is suggested that their nutritional status may be related to the degree and speed of pain relief. In the future, it can be explored whether nutritional interventions targeting picky eating habits can help reduce the frequency of ulcer attacks or alleviate acute phase pain (28). When children's eating is affected by pain, nurses may need to pay attention to the emotional reactions of the children and their parents, provide psychological support, confirm their feelings, and give positive feedback and encouragement to help them cope with the distress caused by pain (29). The above comprehensive intervention measures can reduce the risk of children belonging to the high pain level relief trajectory, promote rapid pain relief, and improve the quality of life of children (30). However, this study is an observational design, and the proposed intervention direction is based solely on factor association analysis, rather than verification of intervention effectiveness. In the future, prospective cohort studies or randomized controlled trials can be conducted to further evaluate the actual effectiveness of personalized interventions tailored to these influencing factors in improving the trajectory of pain development in pediatric patients. Meanwhile, the trajectory classification in this study can serve as a baseline for future long-term prognosis research, providing preliminary evidence for predicting recurrence patterns and the risk of chronic pain transition.

### 4.4 Limitations

Firstly, this study used single center convenience sampling, and the sample was only sourced from our hospital, which limited the extrapolation of the research results. Secondly, pain scores are retrospectively extracted based on medical records. The four evaluation time points (T0-T3) are not completely standardized fixed calendar date follow-up for all patients, but are extracted based on the clinical visit time points recorded in the medical records, which may result in information bias. Moreover, the degree of pain reported by the child or parents lacks objective verification, which may lead to recall bias or reporting bias. Again, the pain dimension of the oral ulcer local sign score used in this study is a descriptive grading scale with a score of 0–3 points. Although it has clinical practicality in the field of oral mucosal diseases, this tool is not a validated pediatric specific pain scale, and the grading is relatively rough and does not cover the emotional dimension of pain. In addition, the main analysis treated pain scores as a continuous variable. Although the robustness of the results was verified through sensitivity analysis of ordered classification, and the use of “whether it affects eating” as a distinguishing criterion for each level approximately met the equidistant hypothesis, this treatment method may still introduce bias. Meanwhile, this study did not standardize records or adjust for confounding factors regarding treatment exposure during follow-up, such as frequency and dosage of local anesthetics and anti-inflammatory mouthwashes. Although the variable of “whether hormones/antibiotics were used” was collected and univariate analysis showed no difference between groups, residual confounding may still exist. Moreover, although the 14 day observation period can cover the complete course of RAS from acute onset to basic healing, it cannot infer the long-term trend of recurrence frequency. Finally, this study did not conduct a mixed model analysis of conditional latent variable growth. Future research may consider expanding the sample source to include pediatric patients from multiple regional hospitals, using prospective cohort studies and standardized pediatric pain scales for evaluation, combined with biomarkers, and detailed recording of treatment exposure to improve the representativeness and reliability of the study results.

## 5 Conclusion

This study found that there is a certain heterogeneity in the development trajectory of pain levels in children with RAS, which can be divided into three potential categories: high pain level relief type, moderate pain level improvement type, and low pain level stable type. Analysis of influencing factors shows that for high pain relief type, ulcer size, number of ulcers, ulcer type, frequency of attacks, and picky eating are independent influencing factors. For the moderate pain level improvement type, ulcer classification, frequency of attacks, and picky eating are independent influencing factors. The above findings can provide reference for identifying high-risk pain trajectory children and developing personalized management strategies, but the effectiveness of relevant intervention measures still needs further research and verification.

## Data Availability

The datasets presented in this study can be found in online repositories. The names of the repository/repositories and accession number(s) can be found in the article/Supplementary Material.
