# Supplementary material for: Pain severity trajectory and influencing factors in children with recurrent aphthous stomatitis: a latent variable growth mixture model
Source: Front Pain Res (Lausanne). 2026 Jul 7;7:1869523. doi: 10.3389/fpain.2026.1869523 (PMC13384924; doi:10.3389/fpain.2026.1869523)
Supplement: Supplementary file 2 [file Table2.docx]

Supplementary Material

**Sensitivity Analysis Treating Pain Scores as Ordered Categorical Variables**

**I. Objective of the Sensitivity Analysis**

In the main analysis of this study, pain scores (0-3 points) were treated as continuous variables. To examine the robustness of this approach, we treated pain scores as ordered categorical variables, refitted the latent growth mixture model (LGMM) using the WLSMV (weighted least squares mean- and variance-adjusted) estimator in Mplus 8.3, and compared the results with those of the main analysis.

**II. Analytical Methods**

Model specification:

(1) Pain scores (T0, T1, T2, and T3) were declared as ordered categorical variables (CATEGORICAL);

(2) The WLSMV estimator was used;

(3) All other settings were consistent with the main analysis: a linear growth model, time loadings fixed at 0, 3, 7, and 14, and homogeneous within-class variances.

Analytical steps:

(1) Redetermine the optimal number of latent classes;

(2) Compare the trajectory patterns across classes;

(3) Repeat the multivariable logistic regression analysis.

**III. Sensitivity Analysis Results**

(1) Model selection:

Models with 1 to 5 latent classes were estimated using the WLSMV estimator. The fit indices are shown in Table 1.

Table 1. Latent classes of pain severity trajectories in children with recurrent oral ulcers

| Number of classes | Entropy | LRT | BLRT | Class proportions |
| --- | --- | --- | --- | --- |
| 1 |  |  |  |  |
| 2 | 0.851 | 0.038 | 0.035 | 0.43/0.57 |
| 3 | 0.831 | 0.024 | 0.021 | 0.39/0.36/0.25 |
| 4 | 0.798 | 0.118 | 0.108 | 0.33/0.29/0.23/0.15 |
| 5 | 0.775 | 0.356 | 0.342 | 0.30/0.27/0.21/0.14/0.08 |

Note: The WLSMV estimator does not directly provide AIC, BIC, or aBIC values.

The results showed that when the number of classes was 3, Entropy = 0.831 > 0.800, BLRT = 0.021 < 0.05, and the proportion of each class was greater than 10%. When the number of classes was 4, BLRT = 0.108 > 0.05, indicating that the four-class model was not significantly superior to the three-class model. Therefore, the three-class model remained supported as the optimal solution, consistent with the main analysis.

(2) Comparison of trajectory patterns:

Based on the three-class model, the mean pain scores for each class at each time point are shown in Table 2.

Table 2. Mean pain scores by class in the ordered categorical variable analysis

| Latent class | T0 | T1 | T2 | T3 | Class proportion (%) |
| --- | --- | --- | --- | --- | --- |
| High pain level remission type | 2.1 | 1.7 | 1.2 | 0.8 | 39 |
| Moderate pain level improvement type | 1.6 | 1.3 | 0.9 | 0.6 | 36 |
| Low pain level stable type | 1.0 | 0.8 | 0.5 | 0.3 | 25 |

Comparison with the main analysis:

Class 1 (high pain level remission type): main analysis intercept 2.2 vs. sensitivity analysis 2.1

Class 2 (moderate pain level improvement type): main analysis intercept 1.6 vs. sensitivity analysis 1.6

Class 3 (low pain level stable type): main analysis intercept 1.0 vs. sensitivity analysis 1.0

The class proportions were generally consistent with those in the main analysis (41%/34%/25%).

Conclusion: The trajectory patterns of all classes were generally consistent with those of the main analysis.

(3) Estimated growth parameters:

Table 3. Estimated growth parameters for each latent class in the ordered categorical variable analysis

| Latent class | Mean intercept (initial pain, points) | Intercept variance | Mean slope (rate of change, points/day) | Slope variance | Mean posterior probability | Class proportion (%) |
| --- | --- | --- | --- | --- | --- | --- |
| High pain level remission type | 2.10 (0.11) | 0.42 (0.07) | -0.098 (0.014) | 0.03 (0.01) | 0.91 | 39.00 |
| Moderate pain level improvement type | 1.60 (0.09) | 0.30 (0.06) | -0.072 (0.011) | 0.02 (0.01) | 0.88 | 36.00 |
| Low pain level stable type | 1.00 (0.07) | 0.20 (0.05) | -0.046 (0.009) | 0.01 (0.00) | 0.93 | 25.00 |

Note: Standard errors are shown in parentheses. All slopes were statistically significant (P < 0.001). Compared with Table 2 of the main analysis, the parameter estimates were highly consistent, with all differences within 0.1.

(4) Multivariable logistic regression analysis:

Using the low pain level stable type as the reference group, the multivariable logistic regression analysis was repeated. The results are shown in Table 4.

Table 4. Multivariable logistic regression results from the ordered categorical variable analysis

| Influencing factor | High pain level remission type OR (95% CI) | P | Moderate pain level improvement type OR (95% CI) | P |
| --- | --- | --- | --- | --- |
| Ulcer size (<5 mm vs. >=5 mm) | 0.261 (0.098, 0.695) | 0.007 | 0.512 (0.208, 1.260) | 0.145 |
| Number of ulcers (1-2 vs. >=3) | 0.248 (0.090, 0.683) | 0.006 | 0.489 (0.196, 1.220) | 0.124 |
| Ulcer type (minor vs. severe/herpetiform) | 0.172 (0.060, 0.492) | 0.001 | 0.342 (0.130, 0.899) | 0.028 |
| Attack frequency (<=6 times/year vs. >6 times/year) | 0.195 (0.075, 0.507) | <0.001 | 0.342 (0.130, 0.899) | 0.044 |
| Picky eating (no vs. yes) | 0.224 (0.088, 0.570) | 0.002 | 0.438 (0.196, 0.978) | 0.043 |

Note: The reference groups were ulcer size >=5 mm, number of ulcers >=3, severe/herpetiform ulcer type, attack frequency >6 times/year, and picky eating.

Comparison with the main analysis:

The direction of all OR values was consistent with the main analysis (all <1, indicating protective factors).

The significance levels were consistent with the main analysis: in the high pain type, all five variables had P < 0.01; in the moderate pain improvement type, ulcer type, attack frequency, and picky eating had P < 0.05, whereas ulcer size and number of ulcers had P > 0.05.

The 95% confidence intervals of the OR values overlapped substantially with those in the main analysis.

**IV. Conclusion of the Sensitivity Analysis**

In summary, the results of the ordered categorical variable sensitivity analysis were highly consistent with those of the main analysis:

(1) Model selection: the three-class model remained supported as the optimal solution;

(2) Trajectory patterns: the trajectory shapes, intercepts, and slopes of all classes were generally consistent with those of the main analysis;

(3) Influencing factors: the direction of the OR values and the significance levels of all variables were consistent with those of the main analysis.

These findings indicate that the main conclusions of this study are robust to the method used to measure the variables.

**V. Summary Comparison of the Main Analysis and Sensitivity Analysis Results**

Table 5. Summary comparison of key results between the main analysis and sensitivity analysis

| Comparison item | Main analysis (continuous variable, MLR) | Sensitivity analysis (ordered categorical, WLSMV) | Consistency |
| --- | --- | --- | --- |
| Optimal number of classes | 3 classes | 3 classes | Consistent |
| Entropy | 0.846 | 0.831 | Consistent |
| High pain type intercept | 2.20 | 2.10 | Consistent |
| Moderate pain type intercept | 1.60 | 1.60 | Consistent |
| Low pain type intercept | 1.00 | 1.00 | Consistent |
| High pain type slope (points/day) | -0.105 | -0.098 | Consistent |
| Moderate pain type slope (points/day) | -0.075 | -0.072 | Consistent |
| Low pain type slope (points/day) | -0.048 | -0.046 | Consistent |
| Influencing factors for high pain type | All 5 significant | All 5 significant | Consistent |
| Influencing factors for moderate pain type | 3 significant | 3 significant | Consistent |
